# Temporal-Spatial Neural Activation Patterns Linked to Perceptual Encoding of Emotional Salience

**DOI:** 10.1371/journal.pone.0093753

**Published:** 2014-04-11

**Authors:** Rebecca M. Todd, Margot J. Taylor, Amanda Robertson, Daniel B. Cassel, Sam M. Doesberg, Daniel H. Lee, Pang N. Shek, Elizabeth W. Pang

**Affiliations:** 1 Department of Psychology, University of British Columbia, Vancouver, British Columbia, Canada; 2 Department of Diagnostic Imaging, Hospital for Sick Children, Toronto, Ontario, Canada; 3 Neurosciences and Mental Health Research Institute, Hospital for Sick Children, Toronto, Ontario, Canada; 4 Department of Psychology, University of Toronto, Toronto, Ontario, Canada; 5 Department of Medical Imaging, University of Toronto, Toronto, Ontario, Canada; 6 Military Medicine Section, Defence Research and Development, Toronto, Ontario, Canada; 7 Division of Neurology, Hospital for Sick Children, Toronto, Ontario, Canada; Harvard Medical School/Massachusetts General Hospital, United States of America

## Abstract

It is well known that we continuously filter incoming sensory information, selectively allocating attention to what is important while suppressing distracting or irrelevant information. Yet questions remain about spatiotemporal patterns of neural processes underlying attentional biases toward emotionally significant aspects of the world. One index of affectively biased attention is an emotional variant of an attentional blink (AB) paradigm, which reveals enhanced perceptual encoding for emotionally salient over neutral stimuli under conditions of limited executive attention. The present study took advantage of the high spatial and temporal resolution of magnetoencephalography (MEG) to investigate neural activation related to emotional and neutral targets in an AB task. MEG data were collected while participants performed a rapid stimulus visual presentation task in which two target stimuli were embedded in a stream of distractor words. The first target (T1) was a number and the second (T2) either an emotionally salient or neutral word. Behavioural results replicated previous findings of greater accuracy for emotionally salient than neutral T2 words. MEG source analyses showed that activation in orbitofrontal cortex, characterized by greater power in the theta and alpha bands, and dorsolateral prefrontal activation were associated with successful perceptual encoding of emotionally salient relative to neutral words. These effects were observed between 250 and 550 ms, latencies associated with discrimination of perceived from unperceived stimuli. These data suggest that important nodes of both emotional salience and frontoparietal executive systems are associated with the emotional modulation of the attentional blink.

## Introduction

Emotionally important events are perceived more vividly than mundane ones [1], and emotionally compelling objects in the environment capture the eye as we navigate the world [2], [3]. It is well known that we continuously filter incoming sensory information, selectively allocating attention to what is important to us and suppressing distracting or irrelevant information (for review see [4]). Yet questions remain about spatio-temporal patterns of neural processes underlying attentional biases toward emotionally significant aspects of the world.

A wide body of research indicates that enhanced visual processing of goal-relevant stimuli is modulated by executive attentional processes in the service of explicit goals [5], [6], [7]. Evidence from affective science suggests that selective visual attention is also modulated by emotional or motivational salience linked to longer-term subjective goals of increasing pleasure and avoiding pain [8], [9]. Such long-term goals can tune visual attention habitually to emotionally salient stimuli such as a prized possession, a facial expression, or a gruesome scene [8], [10], [11]. Convergent data suggest that the amygdalae, orbitofrontal cortices, and other brain regions key in tagging emotional salience may underlie deployment of *affect-biased attention* by modulating visual cortical activation in much the same way as frontoparietal regions do during task-based control [12], [13], [14], [15].

Convergent evidence suggests that, at a latency sufficiently late to allow awareness and explicit evaluation of stimuli, both executive and emotional salience systems contribute to enhanced processing of emotional stimuli. ERP studies have identified a late positive component, the late positive potential (LPP), between 400 and 600 ms after stimulus onset, which is enhanced for emotionally salient over neutral stimuli (e.g., [16]). A recent study found that LPP amplitude for emotionally evocative images can be modulated by both task relevance (executive top-down attention) as well as by the emotional salience of an image when it is not task relevant [17]. This finding suggests that, at that latency, executive and emotional salience systems interact to modulate cortical activation. Building on this finding, one magnetoencephalography (MEG) study found that in the 400–500 ms time window, frontal and occipitoparietal regions showed greater bidirectional activation for more emotionally salient stimuli [18]. Again, this finding was interpreted as reflecting interaction between perceptual and reflective processes. These studies suggest that executive and emotional attention systems may work in an additive manner for later stage processing. A further question concerns whether similar patterns of co-activation between executive and affect-biased attention can be observed for rapid attentional capture by emotional stimuli.

A behavioural index of affect-biased attention that may tap more directly the deployment of emotional control settings is an emotional variant of an attentional blink (AB) paradigm. The AB [19] is a well-documented experimental manipulation that effectively measures biases in perceptual encoding and resulting awareness. The AB task is a rapid serial visual presentation (RSVP) task in which target stimuli are embedded in a stream of very rapidly presented stimuli. The ‘blink’ itself is a phenomenon where participants are typically unable to report a target stimulus (T2) when it is presented within ∼500 ms of a previous target (T1) within the stream of distractors. ERP research has shown that, whereas perceptual components in the first 150–200 ms of stimulus onset are identical for both perceived and unperceived targets, an unperceived T2 stimulus fails to elicit a P3 component [20], [21], which is measured at a similar latency to the LPP reported in emotional studies. fMRI studies implicate frontoparietal networks in the AB, likely functioning in interaction with visual cortices (for review see [22]). Moreover, MEG research has revealed ‘blink sparing’ for perceived T2s to be characterized by greater synchronization between frontoparietal regions at ∼400 within the gamma frequency range [23].

There are a number of interpretations of this phenomenon. One influential interpretation proposes that the blink occurs due to limitations in executive attention involved in the later stage of perceptual encoding, which is required for stimulus awareness, as attention is still caught up in processing the first target. Attentional resources are thus unavailable for processing the second target [24]. Another interpretation holds that the blink reflects a failure to switch attentional sets from those tuned to the category of the T1 stimulus to those tuned to the T2 stimulus if T2 appears too quickly after T1, resulting in a lack of perceptual awareness of T2 [25]. A current review of the literature concludes that executive resources as well as stimulus relevance influence the AB [22].

Notably, when T2 is emotionally salient, a reduced blink, or an emotional sparing, is observed [26], [27], [28]. A recent ERP study with emotional faces as T2 stimuli employed trial by trial analysis of AB effects [29]. This study found a late pattern of activation that was both more accurate and fine-grained in its discrimination of T2 expression. This late latency activity is consistent with models proposing contributions of both executive attention and emotional salience to later activation patterns associated with AB sparing. The same study also found rapid perceptual differentiation between emotionally expressive and neutral faces [29]. Taken with other findings of cortical activation for emotionally salient T2s at 120–240 ms [30], there is some evidence that rapid facilitation of perceptual processing also characterizes emotional sparing

fMRI studies have further suggested that the amygdalae and prefrontal regions are implicated in emotional sparing of the AB, although designs have been varied and results mixed. A study using emotional faces found greater dorsal anterior cingulate activation for perceived vs. unperceived emotional vs. neutral T2s [31]. Another study found both orbitofrontal and amygdala regions to be associated with emotional sparing for T2 [32]. Finally, A recent study using an aversively conditioned T2 found that, although enhanced amygdala and visual cortex activation are implicated in emotional sparing, the relation between them was statistically mediated by frontal activation [33].

These findings suggest that, while key regions of both executive and emotional salience systems are implicated in emotional sparing, increased frontoparietal activation ultimately may drive amygdala facilitation of perceptual encoding. However, the latency at which the amygdalae or other emotional salience hubs, in comparison with frontoparietal regions, influence emotional sparing is still not known. The excellent spatiotemporal resolution of MEG is well suited to map the temporal sequence by which specific regions contribute to emotional sparing. For example, beamformer analysis of M/EEG data has contributed substantially to our understanding of the neurodynamics of selective spatial attention by imaging reliable sequences of cortical activation [34], [35] Although there has been some controversy about the reliability of amygdala activation detected with MEG, a sufficient number of studies have demonstrated reliable activation in the amygdalae using beamforming techniques to indicate that results are informative (e.g., [36], [37], [38], [39]). Moreover, some MEG studies have found activation in the region of the amygdalae to be very rapid (as early as 60 ms), preceding emotionally enhanced visual activation [13], [40], [41]. In the present study, we examined whether relatively rapid amygdala activation might also precede prefrontal activation to contribute to blink sparing.

In summary, whereas fMRI studies have elucidated brain regions implicated in emotional sparing, and ERP studies have suggested both rapid and extended correlates, to date no study has employed MEG in an emotional AB paradigm to obtain both spatial and temporal information with the same protocol. The present study had two major goals: 1) to use the spatial resolution of MEG to accurately localize the neurophysiological generators underlying emotional sparing of the attentional blink, and 2) to investigate the timing and spectral content of neurophysiological activation underlying emotional sparing. Evoked and oscillatory responses in particular frequency ranges are associated with specific processes underlying neurophysiological information processing (see [42] for review). These oscillations, together with their measurement using M/EEG in the context of cognitive functions, are typically focused on the theta, alpha, beta and gamma frequency ranges [42], [43]. Accordingly, we focused our analyses, as well as our frequency ranges of interest, on commensurate frequency bands. Based on previous research, we were particularly interested in the time course/frequency range of potentially enhanced amygdala and ventral prefrontal activation for emotional targets, and the role of these regions in tagging the emotional salience of a stimulus. We hypothesized that, while nodes of both emotional salience and executive systems would be implicated in emotional sparing, activation in the amygdala would precede activation in regions mediating executive processes.

## Methods

### Participants

Participants were 24 healthy young adults between the ages of 22 and 32 years (mean age 25 yrs). By self-report, all participants had no history of neurological, psychological or psychiatric diseases. All participants had normal or corrected-to-normal vision and were free of retaining wires, braces or other metal on the body as per standard MRI/MEG exclusions. Six participants were excluded from analysis due to: corrupted MEG files (2), missing MRI scans (1), non-completion of the task (1), and excessively low accuracy (<40% correct) (2), leaving a final N of 18 (9 female, aged 20–29, mean age 26). All subjects gave informed consent.

### Ethics Statement

This study was approved by the Hospital for Sick Children Research Ethics Board.

### Experimental task

**Figure 1:**
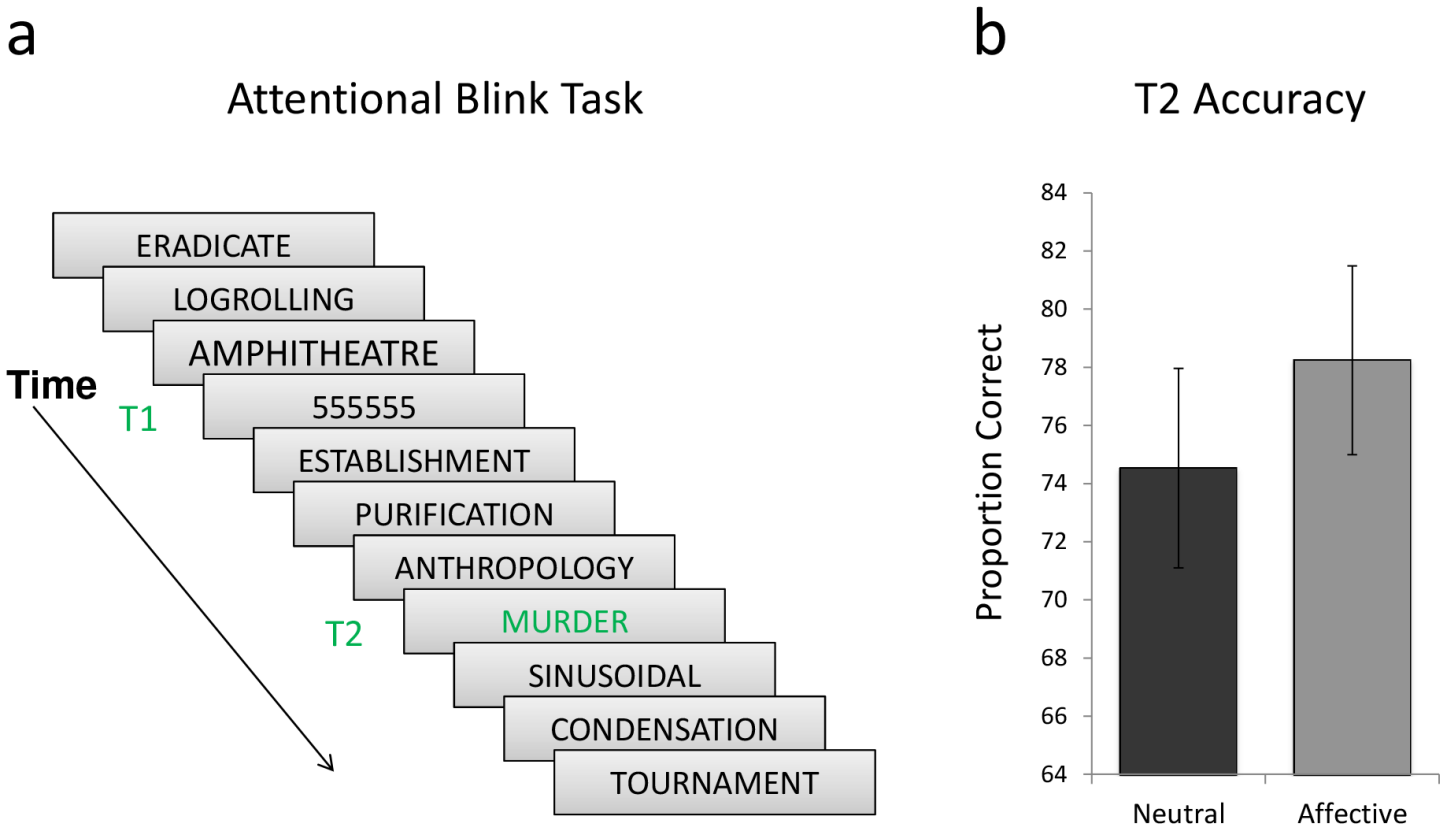
Example of the attentional blink task and behavioral results. a) Within a rapid stream of stimuli occurring every 100 ms, participants must identify two target words (in green). For each subject, T2 occurred at one of 4 possible lags (200, 400, 600 or 700 ms) after the presentation of T1. The lag for each individual, was established using a practice task prior to the MEG task, and was based on the lag at which ∼65% accuracy was achieved. b) Accuracy for emotionally neutral and emotionally salient T2 words.

Before entering the MEG scanner participants performed a practice version of the attentional blink task on a computer. In this rapid serial visual presentation (RSVP) task, in each trial a series of 15 stimuli was presented for 100 ms each in a rapid stream (see Figure 1). Two targets were presented amongst a series of distractors: the first (T1) was a string of numbers and in this practice version the second (T2) was always an emotionally neutral word. Distractor words were emotionally neutral, presented in black font, and selected to be longer than target words to optimize masking effects. T2 stimuli were presented in green font. T2 stimuli were selected from the Emotional Norms for English Words (ANEW), a database of emotionally salient words that have been normatively rated for valence and arousal [44]. T1was randomly positioned in the RSVP stream. T2 followed T1 after one of four possible lags with 0 (lag 1), 1 (lag 2), 3 (lag 4), or 6 (lag 7) distractor words in between. Following each trial, participants were asked to type both targets.

The practice task was used to determine the lag at which each participant had approximately 65% accuracy, and this lag was employed in the MEG task for that participant. Following the practice, the participant completed the attentional blink task again in the MEG where stimuli were presented at midline and subtended a visual angle of 6°. The MEG version of this task was identical to the practice task except that T2 words were either negatively arousing (emotionally salient) or neutral words that were not included in the practice task. Emotionally salient and neutral words were balanced for length, written frequency, and neighbourhood frequency (the frequency of words of the same length that could be created by changing a single letter) [45]. T2 was presented at each subject's determined lag to maximize the number of trials per condition. For each trial, subjects saw a fixation cross for 500 ms, then 15 words were presented at 100 ms intervals. Within this stream of words, following between 2 and 5 distractors, T1 and T2 were presented with the determined lag between them. There was a variable inter-stimulus interval between the final word and the fixation cross during which time the subjects observed a black screen and reported the targets verbally into a microphone. Responses were scored as hits (accurately reported numbers and words) or misses by an experimenter during data acquisition.

### MEG data acquisition

MEG data were recorded continuously (600 Hz sampling rate, DC-100 Hz low-pass filter, third-order spatial gradient noise cancellation), using a 151 channel CTF system (MISL Ltd., Canada) in a magnetically shielded room located at the Hospital for Sick Children, Toronto. Head position within the MEG dewar was determined by the use of three localization coils placed at the nasion and left and right pre-auricular points and measured before and after each run. Conditions with head movements greater than 5 mm are systematically re-run; however, this was not necessary in this study. To co-register MEG data with anatomical images, the 3 localization coils were replaced with MRI-visible contrast markers, and a structural magnetic resonance image (MRI) was obtained from each participant. These images were acquired using T1-weighted MRI scans covering the whole brain (3D MPRAGE (Sag; FOV = 192×240×256 mm; 1 mm iso voxels; TR/TE/TI/FA = 2300/2.96/900/9, GRAPPA = 2) on a 3T MAGNETOM Tim Trio (Siemens AG, Erlangen, Germany) with a 12-channel head coil.

### MEG Analyses

Data pre-processing was completed for each participant as follows: Data were bandpass filtered off-line (zero-phase shifting Butterworth filter) from 1 to 40 Hz, time-locked to T2 stimulus onset and segmented into epochs from 200 ms pre- to 700 ms post-T2. Filtering was optimized for investigation of MEG responses in frequency ranges relevant for cortical processing underlying cognition (theta, alpha, beta, gamma; see [42], [43] for reviews), and specifically associated with attentional blink effects [23], [46], [47]. Trials were binned into emotionally salient hits and neutral hits (where participants correctly identified both targets), and emotionally salient misses and neutral misses (where participants failed to correctly identify T2 words). Trials were averaged within each category for each subject.

Global-field power (GFP) plots showing the root mean squared power across frontal sensors (n = 35) were calculated from each subject's averaged data. Those GFPs were then averaged and overplotted for correct T2 stimuli (regardless of salient or neutral stimulus type) in which T1 stimuli were also correctly reported (hits) and T2 stimuli that were not correctly reported (misses). This was done separately for trials with emotionally salient and neutral T2s, and served as a global index of neural correlates of the attentional blink effect. Since there were unequal numbers of hit and miss trials, the hit and miss GFPs for each subject were weighted by their respective ratios to the overall number of trials.

A vector beamformer source localization algorithm [48], [49] integrated over 100 ms overlapping time windows from 50–550 ms post-T2 for the emotionally salient and neutral words was applied with a spatial resolution of 5 mm. This resulted in images for 9 time windows (i.e., 50–150, 100–200, 150–250, 200–300, 250–350, 300–400, 350–450, 400–500, 450–550 ms). Multisphere head models were created from each individual's MRI [50]. In order to determine regions showing greater activation for emotionally salient than neutral words in each time window, each participant's beamformer images were normalised into stereotaxic space using Advanced Normalization Tools ANTS, University of Pennsylvania, Philadelphia, PA, America; http://stnava.github.io/ANTs/). They were then submitted to one-sided non-parametric permutation tests (2946 permutations) for the contrast emotionally salient>neutral and corrected for multiple comparisons.

To further probe the activation patterns of regions of interest, we placed ‘virtual sensors’ in locations identified by beamforming, and computed the time course of activation at that location. Regions that were expected to be of interest included locations in frontal cortices and amygdalae.

## Results

### Behavioural results

Accuracy was calculated as the proportion of correctly reported words for T2 trials that followed correct T1 trials in the emotional and neutral conditions. Paired sample T-tests revealed greater accuracy for emotional (mean = 78.2) than neutral (mean = 74.5) T2 hits, *t*(1,16) = 2.67, *p* = 0.02 (Figure 1b).

### Global Field Power (GFP) results for hits vs. misses

**Figure 2:**
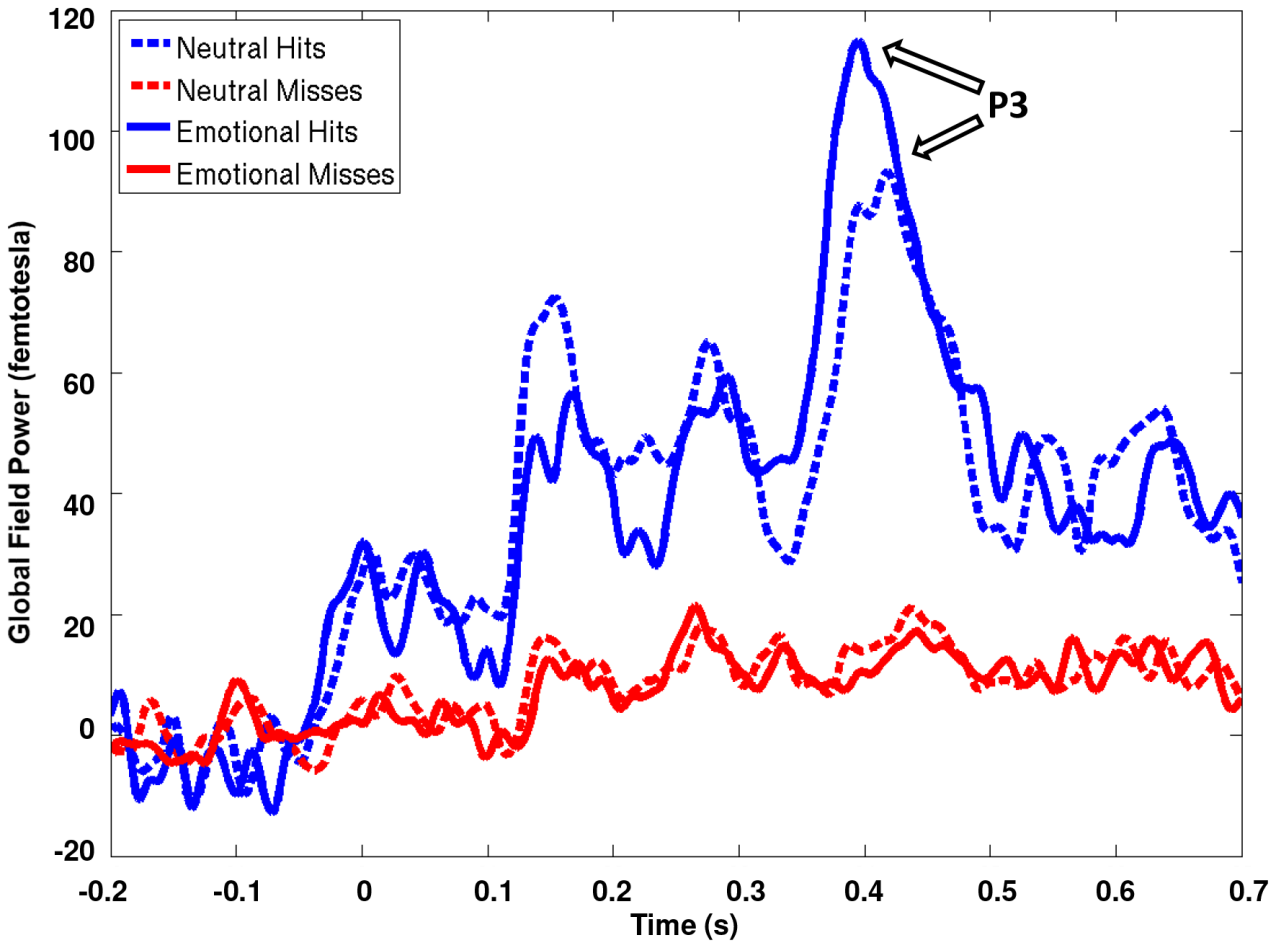
Global field power plots showing the root mean squared power across 35 frontal MEG sensors reveal a P3-like effect for correctly reported T2 words (hits; blue lines) but not for misses (red lines). The P3 effect is present for both neutral (dotted line) and emotional (solid line) words. This finding illustrates previously reported ERP findings that the ‘attentional blink’ effect is reflected in the lack of a P3.

GFP plots from 35 frontal electrodes were visually inspected prior to statistical comparisons of source activation to assess patterns of activation related to task performance across sensors. GFP plots over frontal sensors revealed overall greater activation for accurately reported T2 words that followed correct T1 stimuli (hits), both emotional and neutral, but not for T2 words that were not correctly reported (misses). Consistent with previously reported ERP findings, there was a robust late effect (the P3 in ERPs) for hits and no observable peak for misses for both emotionally salient and neutral words (Figure 2). This replicates previous findings where the P3 has been linked to behavioural indices of awareness (successful perceptual encoding of T2) [51]. There was also a larger P3 peak for emotionally salient than neutral hits.

### Source analyses

**Figure 3:**
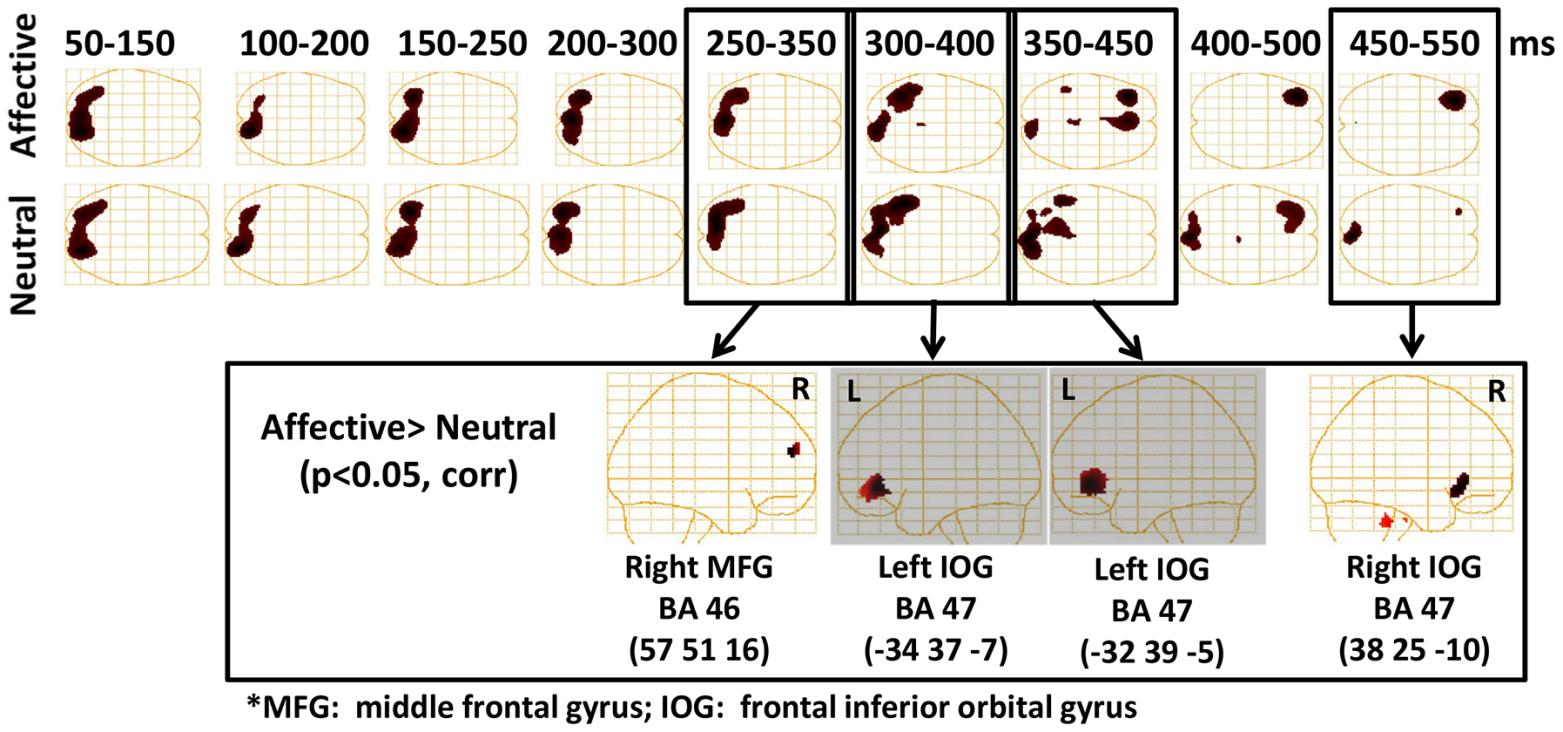
Beamformer source localization results using 100 The top two rows show regions with significant activation for emotional and neutral stimuli respectively in each time window. Image contrasts of the activation between emotional and neutral conditions showed significant greater differences in the right middle frontal gyrus (DLPFC) from 250–350 ms, then in the left inferior orbital gyrus (OFC) for two time windows from 300–400 and 350–450 ms, and then in the right inferior orbital gyrus (OFC) from 450–550 ms.

**Figure 4:**
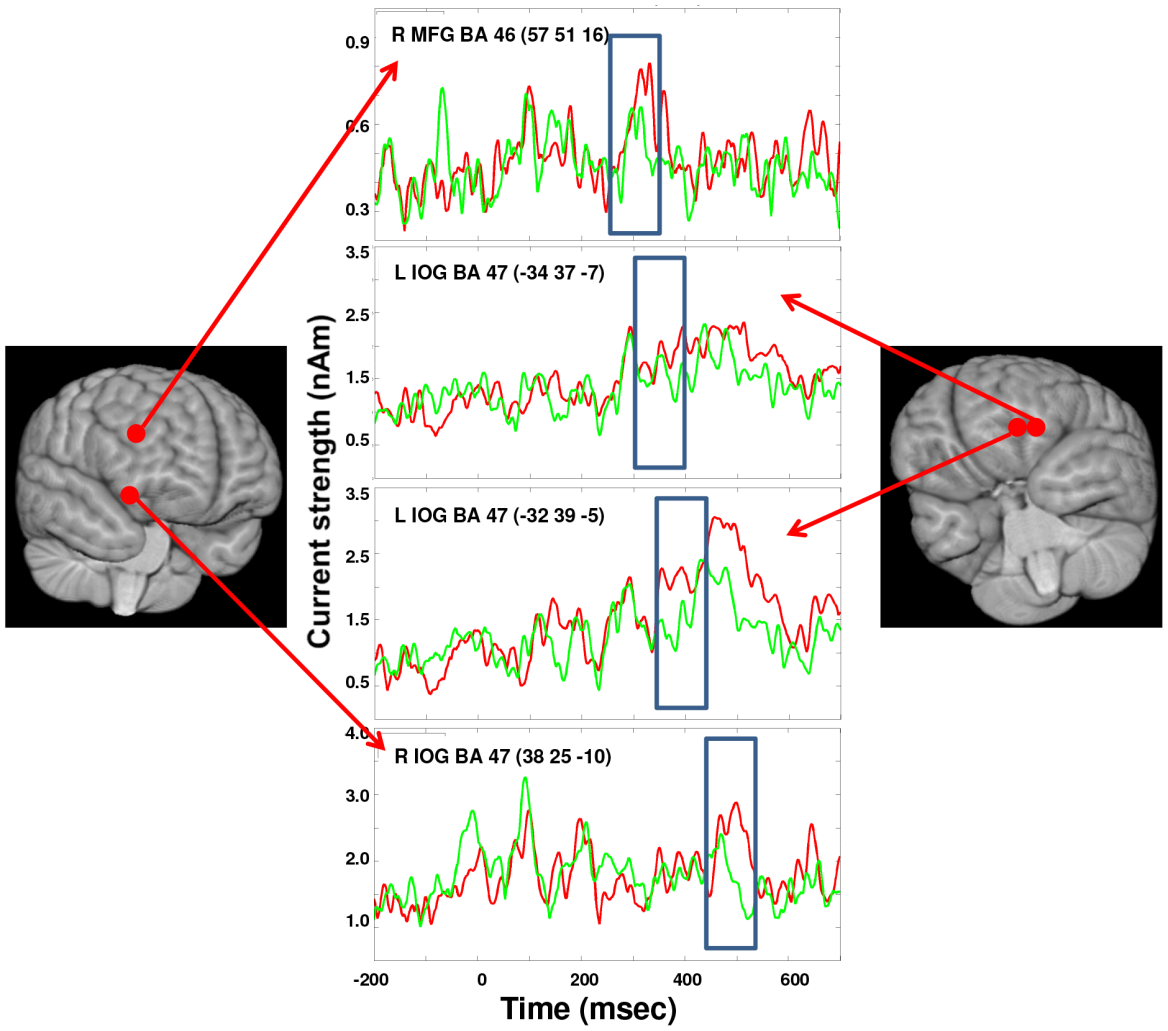
Re-constructed time courses of the right DLPFC and bilateral OFC regions. These were the 4 regions that showed significant greater differences between emotional (red) and neutral (green) stimuli. The time windows where the contrasts were significant are delineated by the blue box. These locations are identified on a 3-dimensional image of the brain.

In order to assess activation across time in specific source regions, we examined emotional and neutral hits separately, relative to a fixation cross baseline, in 100 ms time windows. Results revealed similar patterns of activation in visual cortices at early latencies in both conditions (see Table 1 for all significant activations; *p*<0.05, corrected, t-max). To test for statistically significant activations, data were repeatedly permuted (2946 permutations) across to-be-compared conditions, and the largest differences for any voxel in this surrogate data was used to obtain a threshold for each voxel. Between 350 and 450 ms, at the time of the P3 peak found in the GFP plot, activation was observed in the left middle frontal gyrus in the emotional condition only (Table 1). To directly assess differences between conditions, we completed direct contrasts between emotional and neutral hits. These contrasts revealed four time windows where significantly greater activation (*p*<0.05, corrected for multiple comparisons) was observed for emotional compared to neutral hits (Figure 3). In the 250–350 ms time window there was greater activation for emotional hits in the right dorsolateral prefrontal cortex — specifically the middle frontal gyrus (57, 51, 16, BA 46). In the 300–400 ms and 350–450 ms windows there was greater activation in the left inferior orbital gyrus for emotional hits (peaks at −34, 37, −7, and −32, 39, −5, BA 47, respectively). Finally, in the 450–550 ms window there was significantly greater activation for emotional hits in the right inferior orbital frontal gyrus (38, 25, −10, BA 47). Time courses were re-constructed in these four locations to track the changes in activation to emotional and neutral stimuli (Figure 4). In the pertinent time windows, a clear effect of emotion is seen. Thus, neural differences between emotional and neutral words that were consciously perceived emerged in a region of right dorsolateral prefrontal cortex, followed by left and then right activation in orbitofrontal cortex at the latency of the P3— the time periods in which neural discrimination between hits and misses is observed.

**Table 1:** Beamforming results showing peak activation coordinates for T2 hits relative to a fixation baseline for emotionally salient and neutral words in sliding 100(p<.05, corrected, tmax).

	EMOTIONALLY SALIENT				NEUTRAL			
Time	Talaraich	Anatomical label	BA	Max activ	Talaraich	Anatomical label	BA	Max activ
**50–150**	−24 −76 2	L lingual gyrus	19	0.82	−24 −76 2	L lingual gyrus	19	0.86
	20 −74 −6	R lingual gyrus	18	0.77	22 −76 −8	R lingual gyrus	18	0.79
	16 −70 33	R precuneus	7	0.75				
**100–200**	18 −78 −5	R lingual gyrus	18	0.91	−22 −70 0	L lingual gyrus		0.9
	−22 −68 0	L lingual gyrus	19	0.88	18 −80 −4	R lingual gyrus	18	0.9
**150–250**	−26 −68 0	L lingual gyrus	19	0.8	−30 −70 5	L lingual gyrus	19	0.83
	18 −78 −5	R lingual gyrus	18	0.77	20 −73 22	R precuneus	31	0.8
	18 −73 22	R precuneus	31	0.76				
**200–300**	2 −78 −8	Rcuneus	18	0.71	−30 −71 9	L post cingulate	30	0.74
	−30 −71 9	L post cingulate	30	0.68	−34 −63 −12	L fusiform gyrus	19	0.69
	−34 −63 −12	L fusiform gyrus	19	0.68				
	16 −75 18	R cuneus	18	0.66				
**250–350**	4 −78 −3	R lingual gyrus	18	0.73	−34 −45 −15	L fusiform gyrus	37	0.61
	−38 −59 20	L mid tempo gyrus	39	0.70				
	−34 −55 −9	Lfusiform gyrus	37	0.67				
**300–400**	−38 −58 28	L mid temp gyrus	39	0.67	−40 −45 1	L parahip gyrus	19	0.67
					−38 −55 30	L sup temp gyrus	39	0.66
**350–450**	−36 35 −8	L mid frontal gyrus	11	0.60	6 −83 10	R cuneus	17	0.55
	2 36 26	R ant cingulate	32	0.57				
**400–500**	−34 36 −9	L mid frontal gyrus	11	0.67	4 −83 8	R cuneus	17	0.53
					−24 47 3	L sup frontal gyrus	10	0.49
**450–550**	−32 42 −12	L mid frontal gyrus	11	0.60	4 −81 11	Rcuneus	17	0.52
					−30 46 −7	L mid frontal gyrus	11	0.45

### Time Frequency Analysis

**Figure 5:**
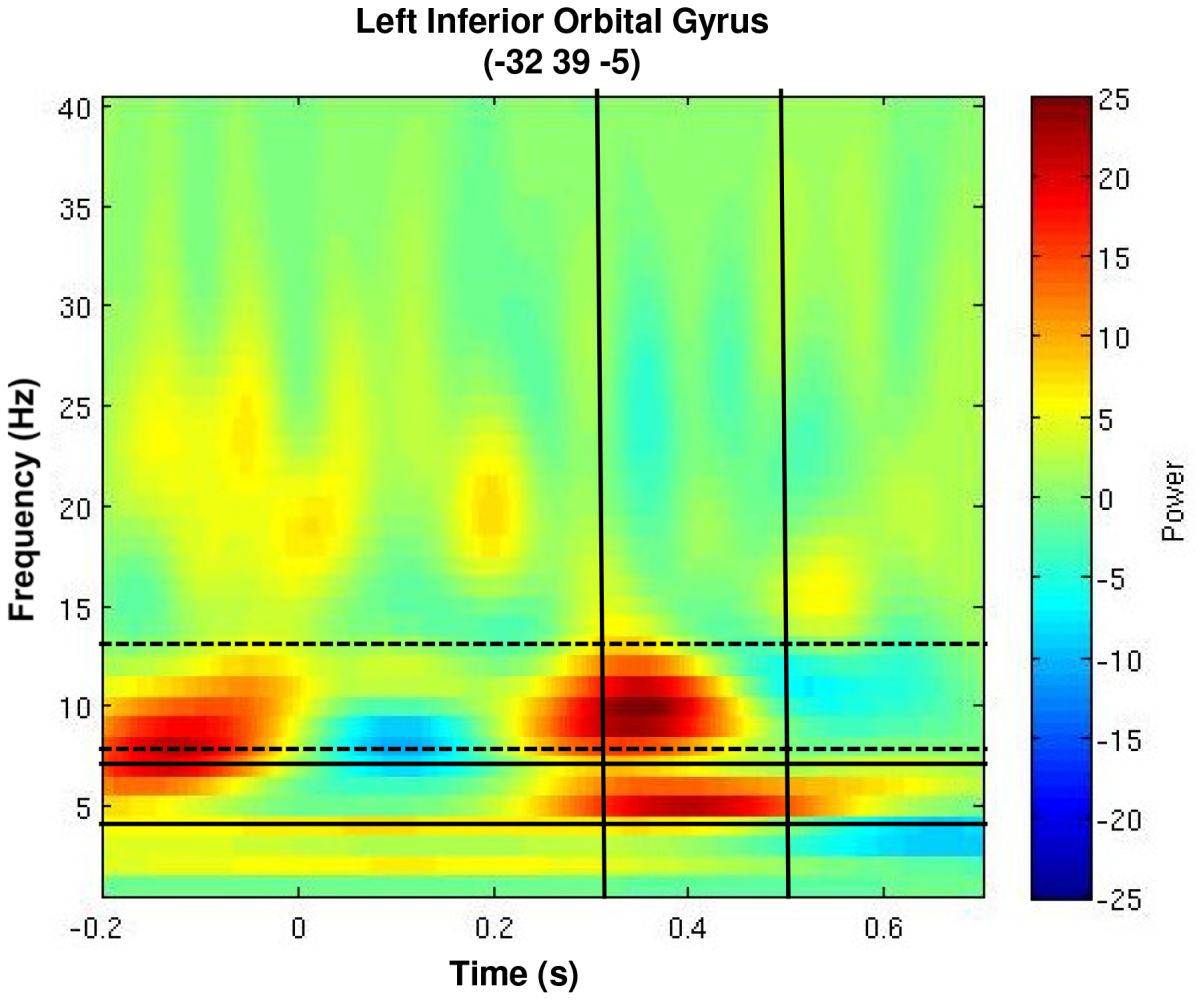
Theta modulation for emotionally salient stimuli in left inferior orbital gyrus (OFC). Time frequency plot reveals strong modulation of theta (solid lines) and alpha (dotted lines) power at this left frontal location between 350–450 ms.

In order to probe specific neural frequencies driving the enhanced left inferior orbital gyrus activation for emotional stimuli between 350 and 450 ms, around the peak latency of the P3 revealed by the GFP, time frequency analysis was performed by reconstructing a broadband time series of activity from each trial using vector beamforming [50]. This algorithm implements a spatial filter to estimate activity at a location in brain space while attenuating contributions from other sources. A time-frequency decomposition was performed on the resulting time series and averaged across trials. T-tests revealed greater summed theta (4–7 Hz) (*t*_(16,one-tailed)_ = 1.54, *p* = 0.04) and alpha (8–13 Hz) (*t*_(16,one-tailed)_ = 1.54, *p* = 0.04) power between 350 and 450 ms for emotional compared to neutral hits (Figure 5). Thus, enhanced left OFC activation for emotional T2 words was primarily driven by greater power in the theta and alpha range.

### Analysis of early peaks in amygdala

**Figure 6:**
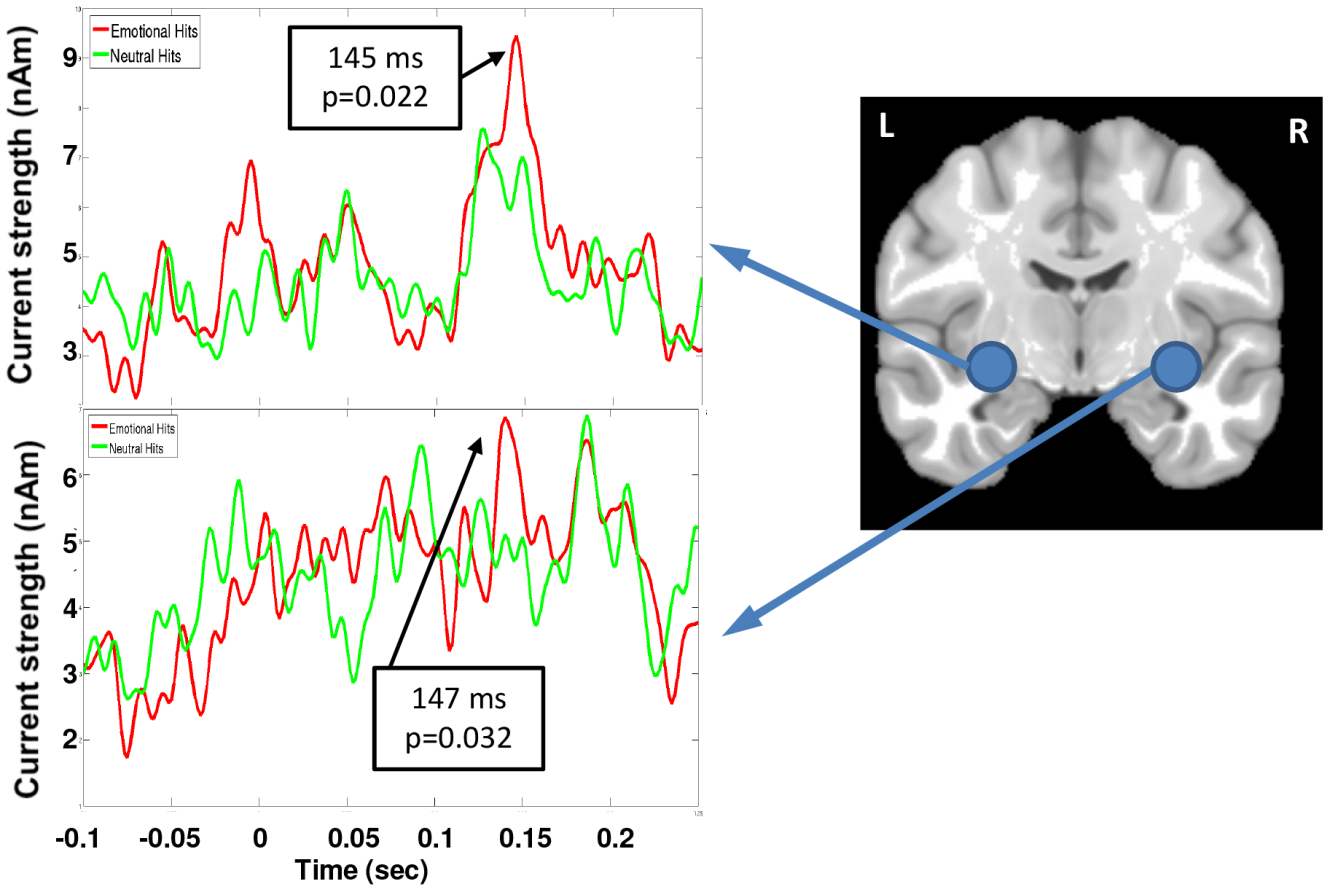
Location of bilateral amygdalae used in the ‘virtual sensor’ analyses are marked and the time-courses of activations for left and right amygdalae are shown with the response to correctly identified emotional stimuli shown in the red tracing and the correctly identified neutral stimuli in the green tracing. The peak latencies where significant differences were seen between the two types of stimuli are marked.

Robust differences between emotion conditions emerged at 250 ms, a latency at which brain activation patterns indexing explicit awareness of targets (P3) also begin to emerge. Yet we also wished to determine regions hypothesized to play a role in selecting which salient stimuli reach awareness. We thus specifically probed brain regions that might show earlier discrimination between emotional and neutral hits, potentially playing a role in initially tagging stimulus salience prior to explicit awareness. We repeated the beamformer analyses using 5 ms non-overlapping time windows between 50–200 ms and contrasted the emotional and neutral conditions to specifically look for potential source locations in the amygdala, as it is expected that this region could be involved in salience tagging. The right amygdala region (32 −2 −10) was identified by these analyses, which suggested activation differences between emotional and neutral stimuli (*p*<0.002, uncorrected). This location was unwarped to each individual's MRI space and ‘virtual sensors’ placed at this coordinate, and in homologous left amygdala (−32 −2 −10), were used to re-construct the time-courses of activation for both types of stimuli (Figure 6). The magnitudes of each subject's individual responses were measured at the latency of the peak averaged response in each amygdala, and these peak magnitudes were submitted to paired t-tests. Activation in the emotional condition was found to be significantly greater (*t*(16,one-tailed) = 1.98, *p* = 0.032) than the neutral condition at 147 ms in the right amygdala. The time course measured in the homologous region of the left amygdala showed a similar peak that was greater for emotional stimuli also at 145 ms (*t*(16,one-tailed) = 2.17, *p* = 0.022). Thus, these follow-up analyses revealed that bilateral amygdala regions showed brief preferential activation for emotionally salient targets at a latency prior to the attentional blink effect (Figure 5).

## Discussion

We investigated the neural underpinnings of the attentional blink using MEG to determine the time course at localized sources as well as the spectral content of brain activation associated with enhanced perceptual encoding of emotionally salient stimuli. Our behavioural data replicated the often-reported ‘emotional sparing’ of the attentional blink, with greater accuracy for emotionally salient than neutral T2 words [26], [27], [28]. For correctly remembered trials, MEG data revealed greater activation for emotionally salient than neutral words in a region of right dorsolateral prefrontal cortex just prior to the peak of the P3 revealed by GFP plots, followed by activation in left orbitofrontal cortex between 300 and 450 ms, around the peak of the P3, which was characterized by greater power in the theta and alpha bands. The left OFC activation was followed closely by activation in right OFC.

Initial inspection of GFP plots revealed a peak activation, maximal at ∼400 ms, which was present for correctly reported T2 words and entirely absent for misses. This pattern of activation is consistent with previous ERP studies finding that, whereas components marking early perceptual processing do not differ between hits and misses, the P3 component (peaking at ∼400 ms) was missing for unperceived targets [21], [51]. Apart from any emotional modulation, the latency of the discrimination of hits from misses is consistent with models of the attentional blink proposing that perceptual categorization at early latencies is unaffected by the processing limitations thought to generate the attentional blink. According to these models, the AB instead reflects a later, resource limited processing stage mediated by frontoparietal networks [20], [24], [51], [52], [53]. Inspection of the GFP also revealed a larger P3-like component for emotionally salient than neutral hits, suggesting that emotional modulation of the blink is also primarily associated with a later processing stage.

Beamformer source localization revealed very similar patterns of early perceptual processing for emotional and neutral hits, in line with previous findings that early perceptual activation is unaffected by attentional blink effects [20], [21], [51]. Activation differences between emotion conditions emerged subsequently in the right dorsolateral prefrontal cortex (DLPFC) followed by sustained activation in bilateral orbitofrontal cortices (OFC). OFC activity showed greater activation for emotionally salient hits in a time window consistent with typically reported P3 effects [52], [54]. The OFC has been implicated in integrating sensory information, influencing autonomic activation, and learning and decision-making processes related to emotional or motivational salience [55]. Data from non-human animal studies indicate a primary role for the OFC in tagging outcome expectancies based in part on motivational value, which depends on an organism's current goals and state [56], [57]. Some OFC neurons have been found to track specifically the acquired salience of a stimulus [58]. Analysis of structural connectivity suggests the OFC provides feedback to sensory cortices linking sensory information to emotional context [59].

Directly prior to the onset of OFC activation, a region of right DLPFC exhibited enhanced processing of successfully encoded emotionally salient stimuli (250–350 ms). The OFC has direct reciprocal anatomical connections with the dorsolateral prefrontal cortex DLPFC, which may facilitate the integration of OFC-mediated processes linked to emotional salience with executive processes subserved by DLPFC [55]. The DLPFC is a key node of frontoparietal executive networks, and supports working memory required for holding targets in mind as well as attentional biasing of visual cortex activation for goal relevant stimuli [5], [60]. Based on convergent evidence, DLPFC has been recently characterized as a node of a multiple demand system, assembling moment to moment attentional content in relation to a hierarchy of goals [61]. Previous MEG studies have shown that DLPFC coupling with occipito-parietal regions has been associated with performance on an emotionally neutral attentional blink task, indicating a role for left DLPFC in executive activity required to successfully distinguish targets from distractors [23]. Such activity includes maintaining an attentional set for the category of target stimuli and comparing it to stimuli in the rapid stream [23]. Moreover, DLPFC activation has further been found be sensitive to emotional salience and mood in tasks requiring working memory [62], [63], suggesting this region also integrates information about emotional salience in tasks that typically activate it. Finally, MEG has revealed enhanced coupling between frontal and occipital regions in the P3/LPP window for emotionally salient images [18]. This finding has been interpreted in terms of reciprocal enhancement of executive attention and emotional salience [18] involved in later stages of processing emotional stimuli.

Our findings are consistent with fMRI data reporting OFC activation as well as frontoparietal activation to be greater for emotionally salient (aversively conditioned) vs. neutral hits in an attentional blink task [33], and with studies suggesting additive effects between affect-biased and executive attention at the time of the P3. Drawing on the spatiotemporal sensitivity of MEG, our results further suggest that the emotional AB taps orbitofrontal and frontoparietal regions associated with both stimulus evaluation and the visual short term memory and elaborative processing required for successful AB performance. Again, this supports findings of additive effects between emotionally biased and executive attention associated with later-stage processing [29], [31]. The combined activation of emotional salience and frontoparietal systems likely contributes to perceptual encoding in relation to both motivational and task-related goals, and to the ‘emotional advantage’ observed for emotionally salient stimuli.

Time-frequency analysis revealed that the enhanced left OFC activation for emotionally salient words between 350 and 450 ms was characterized by increased power in the theta and alpha frequency bands. Increased theta power in the context of the present paradigm is intriguing due to numerous prior EEG/MEG results implicating increases of frontal theta in cognitive and emotional processes (for review see [64]) — consistent with the view that neocortical theta oscillations play an important role in cognitive and perceptual processing [65]. Such modulation of theta in frontal systems appears additionally relevant considering evidence implicating theta in regulating oscillations in other frequency ranges (e.g. [66], [67]) and organizing information flow among cortical regions [68], [69]. Theta-mediated processes have also been associated with perception of visual stimuli [70]. In light of such results, the present observation of increased theta power in the orbitofrontal cortex suggests that this may represent the transfer of information amongst frontal systems mediating perceptual awareness influenced by greater emotional salience in the perceived stimuli.

Increased alpha-band power was also observed in this region during the same 350–450 ms time window. Task-dependent increases in local alpha oscillations have been associated with increasing inhibitory processing [71]. This view is supported by observations from tasks measuring selective visuospatial attention [72], working memory [73], and motor control [74]. However, increased alpha activity does not universally reflect inhibition. For example, visual ERPs understood to index active stimulus processing have been shown to reflect phase resetting of alpha oscillations [75], [76]. This finding is in line with recent reports that alpha oscillations may have multiple generators [77], some of which may be differentially related to cortical activation/deactivation [78]. It is becoming increasingly clear, however that alpha rhythms play an important role in the coordination of information exchange among brain regions [79], [80]. Given this increasingly complex view of the functional role of alpha oscillations, orbitofrontal increases for perceived emotional stimuli are more difficult to interpret than modulations of theta-band oscillations, but may reflect increased activation or transfer of information among frontal regions implicated in perceptual awareness.

Based on previous findings of rapid divergence in ERP activation between hits and misses at 170–200 ms [51], we reasoned that it is likely that initial tagging of stimulus salience occurs prior to the time window in which we observed OFC/DLPFC activation. Exploratory analysis revealed brief amygdala activation peaking at 148 ms that was greater for emotional than neutral stimuli. While these results must be interpreted with caution, they are suggestive and consistent with previous MEG studies of rapid amygdala response to stimulus salience [13], [40], [41]. The amygdalae are densely interconnected with the OFC both structurally and functionally [59], [81]. Projections from the amygdalae to the middle layers of the OFC, which resemble projections from sensory cortices, suggest a pathway of feed-forward salience-related information from the amygdalae to the OFC [59]. In addition, projections from the amygdalae to other regions of prefrontal cortex have been found to be consistent with a role for the amygdalae in focusing frontoparietally-mediated attention on emotionally salient events [81]. If replicated, the present finding would indicate a role for the amygdalae in initial sorting of salient from neutral stimuli that influences successful perceptual encoding, potentially based on emotional control sets tuned by ongoing motivational goals.

Some limitations to the current study should be noted. First, while we filtered the data to focus on cognitive processes implicated in the attentional blink, use of a 1-Hz high-pass filter precludes examination of slow wave activation that has been implicated in emotional processing [18]. Second, use of techniques to probe the directionality of co-activation patterns such as Granger causality analysis (e.g., [18]) could have strengthened our conclusions, and could further explicate patterns of co-activation between key regions.

In summary, the current data indicate that regions implicated in both affect-biased attention and executive processes are associated with the emotional modulation of the attentional blink at latencies characterized by marked differences in activation patterns between perceived and unperceived targets. Thus, the emotional sparing may result from participants attending both differently and more, with perceptual encoding of emotionally salient stimuli benefitting from emotional and executive (task-driven) attentional sets. However there was some indication that activation in the amygdalae precedes greater activation in frontoparietal and OFC regions, suggesting the emotional salience system may be a driving force. Future research can further examine the putative role of amygdala-centred systems as driver by examining phase synchrony between amygdalae, OFC, and other regions in the alpha and theta bands. It can also investigate potential patterns of theta/gamma coupling [66], [67], [70] thought to support moment-to-moment awareness, in an emotional AB paradigm.

## References

[1] Todd RM, Talmi D, Schmitz TW, Susskind J, Anderson AK (2012) Psychophysical and neural evidence for emotion-enhanced perceptual vividness. J Neurosci 32: 11201–11212. 22895705 10.1523/JNEUROSCI.0155-12.2012 PMC3449277

[2] Soares JJ, Ohman A (1993) Backward masking and skin conductance responses after conditioning to nonfeared but fear-relevant stimuli in fearful subjects. Psychophysiol 30: 460–466. 10.1111/j.1469-8986.1993.tb02069.x 8416072

[3] Nielsen SL, Sarason IG (1981) Emotion, personality, and selective attention. J Pers Soc Psychol 41: 945–960.

[4] Cosmelli D (2009) Attending to the stream of consciousness: A methodological challenge. In: Aboitiz F, Cosmelli D, editors. From attention to goal directed behavior. Berlin: Springer-Verlag. pp. 83–103.

[5] Corbetta M, Shulman GL (2002) Control of goal-directed and stimulus-driven attention in the brain. Nat Rev Neurosci 3: 201–215. 11994752 10.1038/nrn755

[6] Fox MD, Snyder AZ, Vincent JL, Corbetta M, Van Essen DC, et al (2005) The human brain is intrinsically organized into dynamic, anticorrelated functional networks. Proc Natl Acad Sci U S A 102: 9673–9678. 15976020 10.1073/pnas.0504136102 PMC1157105

[7] Folk CL, Remington RW, Johnston JC (1992) Involuntary covert orienting is contingent on attentional control settings. J Exp Psychol Hum Percept Perform 18: 1030–1044. 1431742

[8] Pourtois G, Schettino A, Vuilleumier P (2013) Brain mechanisms for emotional influences on perception and attention: what is magic and what is not. Biol Psychol 92: 492–512. 22373657 10.1016/j.biopsycho.2012.02.007

[9] Todd RM, Cunningham WA, Anderson AK, Thompson E (2012) Affect-biased attention as emotion regulation. Trends Cogn Sci 16: 365–372. 22717469 10.1016/j.tics.2012.06.003

[10] Turk DJ, van Bussel K, Brebner JL, Toma AS, Krigolson O, et al (2011) When “it” becomes “mine”: attentional biases triggered by object ownership. J Cogn Neurosci 23: 3725–3733. 21812569 10.1162/jocn_a_00101

[11] Stanisor L, van der Togt C, Pennartz CMA, Roelfsema PR (2013) A unified selection signal for attention and reward in primary visual cortex. Proc Biol Sci 110: 9136–41. 10.1073/pnas.1300117110 PMC3670348 23676276

[12] Rudrauf D, David O, Lachaux JP, Kovach CK, Martinerie J, et al (2008) Rapid interactions between the ventral visual stream and emotion-related structures rely on a two-pathway architecture. J Neurosci 28: 2793–2803. 18337409 10.1523/JNEUROSCI.3476-07.2008 PMC6670659

[13] Gamond L, George N, Lemarechal JD, Hugueville L, Adam C, et al (2011) Early influence of prior experience on face perception. Neuroimage 54: 1415–1426. 20832479 10.1016/j.neuroimage.2010.08.081

[14] Wieser MJ, McTeague LM, Keil A (2012) Competition effects of threatening faces in social anxiety. Emotion 12: 1050–1060. 22390712 10.1037/a0027069 PMC3482481

[15] West GL, Anderson AA, Pratt J (2009) Motivationally significant stimuli show visual prior entry: evidence for attentional capture. J Exp Psychol Hum Percept Perform 35: 1032–1042. 19653747 10.1037/a0014493

[16] Codispoti M, Ferrari V, Junghofer M, Schupp HT (2006) The categorization of natural scenes: brain attention networks revealed by dense sensor ERPs. Neuroimage 32: 583–591. 16750397 10.1016/j.neuroimage.2006.04.180

[17] Ferrari V, Codispoti M, Cardinale R, Bradley MM (2008) Directed and motivated attention during processing of natural scenes. J Cogn Neurosci 20: 1753–1761. 18370595 10.1162/jocn.2008.20121

[18] Moratti S, Saugar C, Strange BA (2011) Prefrontal-occipitoparietal coupling underlies late latency human neuronal responses to emotion. J Neurosci 31: 17278–17286. 22114294 10.1523/JNEUROSCI.2917-11.2011 PMC6623838

[19] Raymond JE, Shapiro KL, Arnell KM (1992) Temporary suppression of visual processing in an RSVP task: an attentional blink? Journal of experimental psychology Human perception and performance 18: 849–860. 1500880 10.1037//0096-1523.18.3.849

[20] Luck SJ, Vogel EK, Shapiro KL (1996) Word meanings can be accessed but not reported during the attentional blink. Nature 383: 616–618. 8857535 10.1038/383616a0

[21] Vogel EK, Luck SJ, Shapiro KL (1998) Electrophysiological evidence for a postperceptual locus of suppression during the attentional blink. J Exp Psychol Hum Percept Perform 24: 1656–1674. 9861716 10.1037//0096-1523.24.6.1656

[22] Martens S, Wyble B (2010) The attentional blink: past, present, and future of a blind spot in perceptual awareness. Neuroscience and biobehavioral reviews 34: 947–957. 20025902 10.1016/j.neubiorev.2009.12.005 PMC2848898

[23] Gross J, Schmitz F, Schnitzler I, Kessler K, Shapiro K, et al (2004) Modulation of long-range neural synchrony reflects temporal limitations of visual attention in humans. Proceedings of the National Academy of Sciences of the United States of America 101: 13050–13055. 15328408 10.1073/pnas.0404944101 PMC516515

[24] Chun MM, Potter MC (1995) A two-stage model for multiple target detection in rapid serial visual presentation. J Exp Psychol Hum Percept Perform 21: 109–127. 7707027 10.1037//0096-1523.21.1.109

[25] Di Lollo V, Kawahara J, Shahab Ghorashi SM, Enns JT (2005) The attentional blink: resource depletion or temporary loss of control? Psychol Res 69: 191–200. 15597184 10.1007/s00426-004-0173-x

[26] Keil A, Ihssen N (2004) Identification facilitation for emotionally arousing verbs during the attentional blink. Emotion 4: 23–35. 15053724 10.1037/1528-3542.4.1.23

[27] Anderson AK (2005) Emotional influences on the attentional dynamics supporting awareness. J Exp Psychol Gen 134: 258–281. 15869349 10.1037/0096-3445.134.2.258

[28] Anderson AK, Phelps EA (2001) Lesions of the human amygdala impair enhanced perception of emotionally salient events. Nature 411: 305–309. 11357132 10.1038/35077083

[29] Zhang D, Luo W, Luo Y (2013) Single-trial ERP analysis reveals facial expression category in a three-stage scheme. Brain Res 1512: 78–88. 23566819 10.1016/j.brainres.2013.03.044

[30] Keil A, Ihssen N, Heim S (2006) Early cortical facilitation for emotionally arousing targets during the attentional blink. BMC Biol 4: 23. 16857054 10.1186/1741-7007-4-23 PMC1559646

[31] Schwabe L, Merz CJ, Walter B, Vaitl D, Wolf OT, et al (2011) Emotional modulation of the attentional blink: the neural structures involved in capturing and holding attention. Neuropsychologia 49: 416–425. 21195103 10.1016/j.neuropsychologia.2010.12.037

[32] De Martino B, Kalisch R, Rees G, Dolan RJ (2009) Enhanced processing of threat stimuli under limited attentional resources. Cereb Cortex 19: 127–133. 18448453 10.1093/cercor/bhn062 PMC2638742

[33] Lim SL, Padmala S, Pessoa L (2009) Segregating the significant from the mundane on a moment-to-moment basis via direct and indirect amygdala contributions. Proc Natl Acad Sci U S A 106: 16841–16846. 19805383 10.1073/pnas.0904551106 PMC2757860

[34] Green JJ, Doesburg SM, Ward LM, McDonald JJ (2011) Electrical neuroimaging of voluntary audiospatial attention: evidence for a supramodal attention control network. J Neurosci 31: 3560–3564. 21389212 10.1523/JNEUROSCI.5758-10.2011 PMC6622799

[35] Green JJ, McDonald JJ (2008) Electrical neuroimaging reveals timing of attentional control activity in human brain. PLoS Biol 6.

[36] Cornwell BR, Baas JM, Johnson L, Holroyd T, Carver FW, et al (2007) Neural responses to auditory stimulus deviance under threat of electric shock revealed by spatially-filtered magnetoencephalography. Neuroimage 37: 282–289. 17566766 10.1016/j.neuroimage.2007.04.055 PMC2717627

[37] Garolera M, Coppola R, Munoz KE, Elvevag B, Carver FW, et al (2007) Amygdala activation in emotional priming: a magnetoencephalogram study. Neuroreport 18: 1449–1453. 17712272 10.1097/WNR.0b013e3282efa253

[38] Luo Q, Holroyd T, Jones M, Hendler T, Blair J (2007) Neural dynamics for facial threat processing as revealed by gamma band synchronization using MEG. Neuroimage 34: 839–847. 17095252 10.1016/j.neuroimage.2006.09.023 PMC1839041

[39] Cornwell BR, Carver FW, Coppola R, Johnson L, Alvarez R, et al (2008) Evoked amygdala responses to negative faces revealed by adaptive MEG beamformers. Brain Res 1244: 103–112. 18930036 10.1016/j.brainres.2008.09.068 PMC2636966

[40] Hung Y, Smith ML, Bayle DJ, Mills T, Cheyne D, et al (2010) Unattended emotional faces elicit early lateralized amygdala-frontal and fusiform activations. Neuroimage 50: 727–733. 20045736 10.1016/j.neuroimage.2009.12.093

[41] Steinberg C, Dobel C, Schupp HT, Kissler J, Elling L, et al (2012) Rapid and highly resolving: emotional evaluation of olfactorily conditioned faces. J Cogn Neurosci 24: 17–27. 21671742 10.1162/jocn_a_00067

[42] Wang XJ (2010) Neurophysiological and computational principles of cortical rhythms in cognition. Physiol Rev 90: 1195–1268. 20664082 10.1152/physrev.00035.2008 PMC2923921

[43] Ward LM (2003) Synchronous neural oscillations and cognitive processes. Trends Cogn Sci 7: 553–559. 14643372 10.1016/j.tics.2003.10.012

[44] Bradley MM, Lang PJ (1999) Emotional norms for English words (ANEW): Instruction manual and emotional ratings. University of Florida.

[45] Coltheart M, Davelaar E, Jonassen JT, Besner D (1977) Access to the internal lexicon. In: Dornic S, editor. Attention and performance VI. New York: Academic press.

[46] Nakatani C, Ito J, Nikolaev AR, Gong P, van Leeuwen C (2005) Phase synchronization analysis of EEG during attentional blink. J Cogn Neurosci 17: 1969–1979. 16356332 10.1162/089892905775008706

[47] Kranczioch C, Debener S, Maye A, Engel AK (2007) Temporal dynamics of access to consciousness in the attentional blink. Neuroimage 37: 947–955. 17629501 10.1016/j.neuroimage.2007.05.044

[48] Quraan MA, Moses SN, Hung Y, Mills T, Taylor MJ (2011) Detection and localization of hippocampal activity using beamformers with MEG: a detailed investigation using simulations and empirical data. Hum Brain Mapp 32: 812–827. 21484951 10.1002/hbm.21068 PMC6870394

[49] Sekihara K, Nagarajan SS, Poeppel D, Marantz A, Miyashita Y (2001) Reconstructing spatio-temporal activities of neural sources using an MEG vector beamformer technique. IEEE Trans Bio-med Eng 48: 760–771. 10.1109/10.930901 11442288

[50] Lalancette M, Quraan M, Cheyne D (2011) Evaluation of multiple-sphere head models for MEG source localization. Phys Med Biol 56: 5621–5635. 21828900 10.1088/0031-9155/56/17/010

[51] Sergent C, Baillet S, Dehaene S (2005) Timing of the brain events underlying access to consciousness during the attentional blink. Nat Neurosci 8: 1391–1400. 16158062 10.1038/nn1549

[52] Marois R, Yi DJ, Chun MM (2004) The neural fate of consciously perceived and missed events in the attentional blink. Neuron 41: 465–472. 14766184 10.1016/s0896-6273(04)00012-1

[53] Dehaene S, Changeux JP, Naccache L, Sackur J, Sergent C (2006) Conscious, preconscious, and subliminal processing: a testable taxonomy. Trends Cogn Sci 10: 204–211. 16603406 10.1016/j.tics.2006.03.007

[54] Todd JJ, Marois R (2004) Capacity limit of visual short-term memory in human posterior parietal cortex. Nature 428: 751–754. 15085133 10.1038/nature02466

[55] Kringelbach ML (2005) The human orbitofrontal cortex: linking reward to hedonic experience. Nature reviews Neuroscience 6: 691–702. 16136173 10.1038/nrn1747

[56] Rudebeck PH, Saunders RC, Prescott AT, Chau LS, Murray EA (2013) Prefrontal mechanisms of behavioral flexibility, emotion regulation and value updating. Nat Neurosci 16: 1140–1145. 23792944 10.1038/nn.3440 PMC3733248

[57] Schoenbaum G, Roesch MR, Stalnaker TA, Takahashi YK (2009) A new perspective on the role of the orbitofrontal cortex in adaptive behaviour. Nat Rev Neurosci 10: 885–892. 19904278 10.1038/nrn2753 PMC2835299

[58] Ogawa M, van der Meer MA, Esber GR, Cerri DH, Stalnaker TA, et al (2013) Risk-responsive orbitofrontal neurons track acquired salience. Neuron 77: 251–258. 23352162 10.1016/j.neuron.2012.11.006 PMC3559000

[59] Barbas H (2007) Specialized elements of orbitofrontal cortex in primates. Ann N Y Acad Sci 1121: 10–32. 17698996 10.1196/annals.1401.015

[60] Desimone R, Duncan J (1995) Neural mechanisms of selective visual attention. Annual Rev Neurosci 18: 193–222. 7605061 10.1146/annurev.ne.18.030195.001205

[61] Duncan J (2013) The structure of cognition: attentional episodes in mind and brain. Neuron 80: 35–50. 24094101 10.1016/j.neuron.2013.09.015 PMC3791406

[62] Perlstein WM, Elbert T, Stenger VA (2002) Dissociation in human prefrontal cortex of emotional influences on working memory-related activity. Proc Natl Acad Sci U S A 99: 1736–1741. 11818573 10.1073/pnas.241650598 PMC122260

[63] Gray JR, Braver TS, Raichle ME (2002) Integration of emotion and cognition in the lateral prefrontal cortex. Proc Natl Acad Sci U S A 99: 4115–4120. 11904454 10.1073/pnas.062381899 PMC122657

[64] Mitchell DJ, McNaughton N, Flanagan D, Kirk IJ (2008) Frontal-midline theta from the perspective of hippocampal “theta”. Progr Neurobiol 86: 156–185. 10.1016/j.pneurobio.2008.09.005 18824212

[65] Kahana MJ, Seelig D, Madsen JR (2001) Theta returns. Curr Opin Neurobiol 11: 739–744. 11741027 10.1016/s0959-4388(01)00278-1

[66] Canolty RT, Edwards E, Dalal SS, Soltani M, Nagarajan SS, et al (2006) High gamma power is phase-locked to theta oscillations in human neocortex. Science 313: 1626–1628. 16973878 10.1126/science.1128115 PMC2628289

[67] Canolty RT, Knight RT (2010) The functional role of cross-frequency coupling. Trends Cogn Sci 14: 506–515. 20932795 10.1016/j.tics.2010.09.001 PMC3359652

[68] Voytek B, D'Esposito M, Crone N, Knight RT (2013) A method for event-related phase/amplitude coupling. Neuroimage 64: 416–424. 22986076 10.1016/j.neuroimage.2012.09.023 PMC3508071

[69] Lisman JE, Jensen O (2013) The theta-gamma neural code. Neuron 77: 1002–1016. 23522038 10.1016/j.neuron.2013.03.007 PMC3648857

[70] Doesburg SM, Green JJ, McDonald JJ, Ward LM (2009) Rhythms of consciousness: binocular rivalry reveals large-scale oscillatory network dynamics mediating visual perception. PLoS ONE 4: e6142. 19582165 10.1371/journal.pone.0006142 PMC2702101

[71] Klimesch W, Sauseng P, Hanslmayr S (2007) EEG alpha oscillations: the inhibition-timing hypothesis. Brain Res Rev 53: 63–88. 16887192 10.1016/j.brainresrev.2006.06.003

[72] Worden MS, Foxe JJ, Wang N, Simpson GV (2000) Anticipatory biasing of visuospatial attention indexed by retinotopically specific alpha-band electroencephalography increases over occipital cortex. J Neurosci 20: RC63. 10704517 10.1523/JNEUROSCI.20-06-j0002.2000 PMC6772495

[73] Jensen O, Gelfand J, Kounios J, Lisman JE (2002) Oscillations in the alpha band (9–12 Hz) increase with memory load during retention in a short-term memory task. Cereb Cortex 12: 877–882. 12122036 10.1093/cercor/12.8.877

[74] Pfurtscheller G, Stancak A Jr, Neuper C (1996) Event-related synchronization (ERS) in the alpha band–an electrophysiological correlate of cortical idling: a review. Int J Psychophysiol 24: 39–46. 8978434 10.1016/s0167-8760(96)00066-9

[75] Hanslmayr S, Aslan A, Staudigl T, Klimesch W, Herrmann CS, et al (2007) Prestimulus oscillations predict visual perception performance between and within subjects. Neuroimage 37: 1465–1473. 17706433 10.1016/j.neuroimage.2007.07.011

[76] Hanslmayr S, Klimesch W, Sauseng P, Gruber W, Doppelmayr M, et al (2007) Alpha phase reset contributes to the generation of ERPs. Cereb Cortex 17: 1–8. 16452640 10.1093/cercor/bhj129

[77] Buffalo EA, Fries P, Landman R, Buschman TJ, Desimone R (2011) Laminar differences in gamma and alpha coherence in the ventral stream. Proc Natl Acad Sci U S A 108: 11262–11267. 21690410 10.1073/pnas.1011284108 PMC3131344

[78] Schmid MC, Singer W, Fries P (2012) Thalamic coordination of cortical communication. Neuron 75: 551–552. 22920248 10.1016/j.neuron.2012.08.009

[79] Palva S, Palva JM (2007) New vistas for alpha-frequency band oscillations. Trends Neurosci 30: 150–158. 17307258 10.1016/j.tins.2007.02.001

[80] Saalmann YB, Pinsk MA, Wang L, Li X, Kastner S (2012) The pulvinar regulates information transmission between cortical areas based on attention demands. Science 337: 753–756. 22879517 10.1126/science.1223082 PMC3714098

[81] Ghashghaei HT, Hilgetag CC, Barbas H (2007) Sequence of information processing for emotions based on the anatomic dialogue between prefrontal cortex and amygdala. Neuroimage 34: 905–923. 17126037 10.1016/j.neuroimage.2006.09.046 PMC2045074

