# Correction: Temporal-Spatial Neural Activation Patterns Linked to Perceptual Encoding of Emotional Salience

**DOI:** 10.1371/journal.pone.0105648

**Published:** 2014-08-08

**Authors:** The PLOS ONE Staff

The fifth author's name is spelled incorrectly. The correct name is: Sam M. Doesburg. The correct citation is: Todd RM, Taylor MJ, Robertson A, Cassel DB, Doesburg SM, et al. (2014) Temporal-Spatial Neural Activation Patterns Linked to Perceptual Encoding of Emotional Salience. PLoS ONE 9(4): e93753. doi:10.1371/journal.pone.0093753

## References

[1] Todd RM, Taylor MJ, Robertson A, Cassel DB, Doesberg SM, et al (2014) Temporal-Spatial Neural Activation Patterns Linked to Perceptual Encoding of Emotional Salience. PLoS ONE 9(4): e93753 doi:10.1371/journal.pone.0093753 24727751 10.1371/journal.pone.0093753 PMC3984101

